# Governing Heritable Human Genome Editing: A Textual History and a Proposal for the Future

**DOI:** 10.1089/crispr.2021.0043

**Published:** 2021-08-16

**Authors:** LeRoy Walters, Robert M. Cook-Deegan, Eli Y. Adashi

**Affiliations:** ^1^ Kennedy Institute of Ethics, Georgetown University, Washington, DC, USA; ^2^ Consortium for Science, Policy, and Outcomes, Arizona State University, Washington, DC, USA; ^3^ Department of Medical Science, Brown University, Providence, Rhode Island, USA.

## Abstract

Heritable human genome editing (HHGE) has become a topic of intense public interest, especially since 2015. In the early 1980s, a related topic—human genetic engineering—was the subject of sustained public discussion. There was particular concern about germline genetic intervention. During the 1980s debate, an advisory committee to the Director of the National Institutes of Health (NIH)—the Recombinant DNA Advisory Committee (RAC)—agreed to provide initial public review of proposals for deliberate introduction of DNA into human beings. In 1984 and 1985, the RAC developed guidelines for research involving DNA transfer into patients. The committee also commented on the possibility of deliberately altering the human germline. We track the textual changes over time in the RAC's response to the possibility of germline genetic intervention in humans. In 2019, the NIH RAC was abolished. New techniques for genome editing, including CRISPR-based techniques, make both somatic and germline alterations much more feasible. These novel capabilities have again raised questions about oversight. We propose the creation of a new structure for the public oversight of proposals to perform HHGE. In parallel with a technical review by a regulatory agency, such proposals should also be publicly evaluated by a presidentially appointed Bioethics Advisory Commission.

## Introduction

Heritable human genome editing (HHGE) has become a topic of intense public interest and international controversy, especially since 2015.^1,2^ A similar debate about introducing genetic changes into humans occurred in the 1980s. Here, we review that earlier history, with particular attention to the specific texts that guided U.S. policy—and with an eye to proposing a public oversight framework for HHGE.

In 1982, human genetic engineering was a major topic of public discussion. During that year, the President's Commission for the Study of Ethical Problems in Medicine and Biomedical and Behavioral Research published a report titled “Splicing Life: A Report on the Social and Ethical Issues of Genetic Engineering with Human Beings.”^3^ Representative Albert Gore, Jr. (D-TN), then chair of the Subcommittee on Investigations and Oversight of the House Committee on Science and Technology, also led a three-day hearing on human genetic engineering in November 1982—a hearing that featured the “Splicing Life” report.^4^

In this article, we follow the decision by the National Institutes of Health (NIH) to conduct public reviews of proposals to transfer recombinant DNA molecules into humans—an intervention that at that time was referred to as “human gene therapy.” That history can be tracked because the debates about NIH policy were matters of public record. We devote particular attention to what an advisory committee to the NIH Director said about deliberate attempts to perform germline genetic interventions. This textual history presages the current debate about what is called HHGE in the September 2020 report of the U.K. Royal Society and the U.S. National Academies of Sciences and Medicine.^5^

## NIH Review of Germline Genetic Alterations: A Textual History

In 1982, at the NIH, the work of the Recombinant DNA Advisory Committee (RAC) was in some ways winding down. Laboratory research using recombinant DNA (rDNA) had proven to be generally safe, thereby reducing fears about the major concern of the late 1970s: the inadvertent release of harmful rDNA or rDNA-containing organisms into the environment. In the early 1980s, attention shifted to the use of rDNA methods for the production of biologicals such as insulin and to the deliberate release of genetically modified organisms into the environment. The “Splicing Life” report suggested a possible new mission for the RAC: reviewing protocols for DNA transfer into humans.

In 1983 and 1984, the novel field of human gene transfer research and the role of the NIH RAC converged. At its meeting on April 11, 1983, the RAC agreed to assist the NIH in responding to the “Splicing Life” report.^6^ An interdisciplinary working group, meeting in June and December 1983, recommended that the RAC accept a role in overseeing the initial stages of human gene transfer research (see pp. 176–177 of Milewski^6^). The working group's proposal was published in the *Federal Register* on January 5, 1984.^7^ According to the proposed plan, the NIH Director, Dr. James B. Wyngaarden, would appoint a RAC subcommittee to perform an initial review of clinical protocols involving human gene transfer. The RAC itself would then review the recommendations of the subcommittee and forward its advice to the NIH Director. At its meeting on February 6, 1984, the RAC endorsed this proposal (see p. 177 of Milewski^6^). In April 1984, the NIH Director concurred, and Dr. Wyngaarden asked the RAC to proceed.^8^

During the summer of 1984, the initial members of the RAC subcommittee were appointed. They included four laboratory scientists, three clinicians, three ethicists, three lawyers, and two public policy experts (see Table 1).^9^ The RAC subcommittee, designated the Working Group on Human Gene Therapy, held its first public meeting on October 12, 1984 (see pp. 177–178 of Milewski^6^).

**Table 1.** Members of the Working Group on Human Gene Therapy, October 1984

W. French Anderson, M.D., National Heart, Lung, and Blood Institute, NIH (Laboratory Scientist)
Judith Areen, J.D., Georgetown University Law Center (Lawyer)
Richard Axel, M.D., Institute of Cancer Research, Columbia University (Laboratory Scientist)
Alexander Capron, LL.B., Law Center, University of Southern California (Lawyer)
Samuel Gorovitz, Ph.D., Department of Philosophy (Ethicist)
James F. Childress, Ph.D., Department of Religious Studies, University of Virginia (Ethicist)
Susan K. Gottesman, Ph.D., National Cancer Institute, NIH (Laboratory Scientist)
Clifford Grobstein, Ph.D., Department of Science, Technology, and Public Affairs, University of California San Diego (Public Policy)
Maurice J. Mahoney, M.D., Department of Human Genetics, Yale University (Clinician)
Robert E. Mitchell, LL.B., Attorney at Law, Norwalk, California (Lawyer)
Arno G. Motulsky, M.D., Department of Medicine, University of Washington (Clinician)
Robert F. Murray, M.D., Division of Medical Genetics, Howard University (Clinician)
Robert F. Rich, Ph.D., School of Urban and Public Affairs, Carnegie-Mellon University (Public Policy)
Harold E. Varmus, M.D., Department of Microbiology, University of California San Francisco (Laboratory Scientist)
LeRoy Walters, Ph.D., Kennedy Institute of Ethics, Georgetown University (Ethicist)

There was a sense of urgency at that initial meeting of the Working Group. In 1980, UCLA clinician-researcher Martin J. Cline had made premature attempts to administer genetically modified cells to two patients: one patient in Israel and a second in Italy.^10^ Several laboratories were also known to be undertaking preclinical studies of gene therapy. During its first meeting, the Working Group discussed a 13-page draft document: “Points to Consider in the Design and Submission of Human Gene Therapy Protocols.”^9^

At its meeting on October 29, 1984, the RAC parent committee reviewed the Working Group's draft and made suggestions for improvement. On November 16, 1984, the Working Group held its second public meeting and produced a revised 11-page draft, “Points to Consider in the Design and Submission of Human *Somatic Cell* Gene Therapy Protocols” (emphasis added).^9^ The addition of the words “somatic cell” clearly conveyed the Working Group's view that germline intervention should not be undertaken at this early stage of research on human gene transfer. The phrase “somatic cell” also echoed a central thesis of the “Splicing Life” report—namely that somatic cell gene therapy was not an intervention to be feared. In fact, somatic cell gene therapy was analogous to bone-marrow transplantation. The reproductive (germline) cells of the recipient would not be affected, at least not deliberately.

NIH Director Wyngaarden approved the revised draft of the Working Group and forwarded it to the *Federal Register*. The “Points to Consider” document was duly published in the January 22, 1985, *Federal Register* with a request for public comment.^11^

At its meeting on April 1, 1985, the Working Group considered the 15 comments it had received on the “Points to Consider.” Most comments focused on minor technical issues. There was, however, one central question that had not thus far been addressed: Would the Working Group and the RAC make any formal statement about germline genetic intervention?

During the lunch break of the meeting on April 1, three members of the Working Group—Susan K. Gottesman, James F. Childress, and Alexander M. Capron—conferred about the germline question. They proposed this text:

A distinction is usually drawn between making genetic changes in somatic cells, the purpose of which is to treat individual patients, and germ-line alterations, which would affect the genes passed on to the offspring of the persons treated. The RAC and its Working Group will not, at present, entertain porposals [sic] for intentional germ-line treatments, but will review and approve somatic-cell proposals that satisfy the points raised in the following document. (Attachment VII, “Minutes of the RAC Working Group on Human Gene Therapy meeting, April 1, 1985”; see Supplementary Material).

At the post-lunch discussion, W. French Anderson suggested that the first words of the text should read “A distinction should be drawn.” He also commented that the second sentence should read as follows:

The RAC and its Working Group will not, at present, entertain proposals for germ-line treatments, but will consider for approval protocols for somatic cell gene therapy.^9^

The Working Group accepted Dr. Anderson's proposed revisions.

Between the meeting on April 1, 1985, of the Working Group and the meeting on May 3, 1985, of the RAC, the text of that key paragraph was further refined. The language describing somatic and germline interventions was expanded, and the word “alterations” was substituted for “treatments.” The revised text read:

A distinction should be drawn between making genetic changes in somatic cells and in germ-line cells. The purpose of somatic cell gene therapy is to treat an individual patient, e.g., by inserting a properly-functioning gene into a patient's bone marrow cells *in vitro* and then reintroducing the cells into the patient's body. In germ-line alterations, a specific attempt is made to introduce genetic changes into the germ (reproductive) cells of an individual, with the aim of changing the set of genes passed on to the individual's offspring. The RAC and its working group will not, at present, entertain proposals for germ-line alterations but will consider for approval protocols involving somatic-cell gene therapy. (see Supplementary Material)

This language was approved by the RAC on May 3, 1985, and was subsequently published in the *Federal Register* on August 19, 1985.^12^

In 1986, the Working Group on Human Gene Therapy was renamed the Human Gene Therapy Subcommittee. During that year, the subcommittee and the RAC reviewed and updated the “Points to Consider.” At its meeting on September 29, 1986, the RAC approved the revisions suggested by the subcommittee. However, the paragraph that discussed germline genetic intervention remained unchanged.^13^

In January 1989, the subcommittee and the RAC reviewed a Preclinical Data Document submitted by W. French Anderson and a gene-marking protocol submitted by Steven A. Rosenberg *et al*.^14^ The time seemed ripe for a review and update of the “Points to Consider.” A special RAC subcommittee was appointed to undertake this review. The subcommittee met on March 31, 1989 (see Supplementary Material). On July 31, 1989, the Human Gene Therapy Subcommittee reviewed the special subcommittee's draft (minutes unavailable).^15^ The draft was published in the *Federal Register* on September 1, 1989, and forwarded to the RAC, which approved the revisions, with a modification of the document title, at its meeting on October 6, 1989. The revised “Points to Consider” were then published in the *Federal Register* on March 1, 1990.^16^ The revised document was also reprinted in the inaugural issue of a new journal, *Human Gene Therapy*.^17^

On the topic of germline genetic intervention, the 1989 version of the “Points to Consider” included no substantive changes to the 1986 version. However, the sequence of the sentences in the critical paragraph was altered, accentuating the RAC's decision not to review proposals involving germline modification (see Supplementary Material). The 1989 revision reads:

The RAC and its Subcommittee will not at present entertain proposals for germ-line alterations but will consider for approval protocols involving somatic cell gene therapy. The purpose of somatic cell gene therapy is to treat an individual patient, e.g., by inserting a properly functioning gene into a patient's somatic cells. In germ-line alterations, a specific attempt is made to introduce genetic changes into the germ (reproductive) cells of an individual, with the aim of changing the set of genes passed on to the individual's offspring.^18^

This text remained unchanged in the “Points to Consider” from 1989 until April 26, 2019, when the RAC's oversight role concluded, and the “Points to Consider” no longer guided researchers proposing to undertake federally funded gene transfer studies.^19^ The unaltered text was last published in the *Federal Register* on March 22, 2016, with the other sections of the “Points to Consider.”^20^

## Historical Context and Implications for the Current Debate

The language crafted by three members of a RAC working group over lunch on April 1, 1985, proved to be remarkably durable and, with minor tweaks, seems to have guided NIH policy on germline intervention from 1985 through 2019. It may be worthwhile to reconstruct the context in which the language was formulated and to ponder the scope of this paragraph.

In 1982 and 1983, the question of human genetic engineering was provoking a policy debate. Despite the reassuring “Splicing Life” report, activists such as Jeremy Rifkin continued to raise alarms about possible misuses of genetic technologies. On June 8, 1983, Rifkin's organization, the Foundation on Economic Trends, published “The Theological Letter Concerning the Moral Arguments Against Genetic Engineering of the Human Germline Cells.”^21^ This letter was signed by 51 religious leaders and theologians, as well as by five natural scientists and a social scientist. On June 10, 1983, Senator Mark O. Hatfield (R-OR) entered the full text of Rifkin's letter into the Congressional Record.^22^ Hatfield also spoke in support of the theological letter, adding:

Recently, I had an enlightening and troubling 3-hour conversation with several of the Nation's top geneticists. It is likely that soon not only genetic corrections—somatic engineering—will be commonplace, but that sex cell gene removal and replacement—will be possible. No one knows the long-range implications of offspring born of eugenically engineered individuals.^23^

As the Working Group on Human Gene Therapy prepared the “Points to Consider” in 1985, it sought to create a safe haven for somatic cell gene transfer research that aimed to treat disease without transmitting genetic changes from parent to child. One way of achieving this goal was to make clear to the public and the press that its guidelines covered only research protocols that involved somatic cells.

The “Points to Consider” were also an effort to extend the RAC's oversight activities from the late 1970s. During that era, both the public and private sectors, with only a few exceptions, complied with NIH's voluntary public oversight role in reviewing laboratory research with recombinant DNA. The RAC's proposed public review of gene transfer protocols involving human participants expanded on that tradition. In effect, as an advisory committee, NIH's RAC was offering to serve as a national Institutional Review Board (IRB) for the initial stage of human gene transfer research. This offer forestalled the creation of a new regulatory agency for the field of human genetics—an option being advocated by several congressional leaders in the mid-1980s.^24^

Several other features of the germline paragraph also merit attention. The paragraph clearly referred to nuclear DNA, not mitochondrial DNA. The current discussion of mitochondrial replacement (MR), also known as mitochondrial donation, was not yet envisioned in 1985. In addition, the paragraph was gently worded. The phrase “at present” suggested that the RAC was not making a statement for all time. Future developments in biomedical research might create new facts on the ground that would justify a re-examination of the “no germline changes” policy. Moreover, the paragraph did not entirely rule out the possibility of germline changes in the reproductive cells of people who receive somatic cell gene transfer for the treatment of their diseases. There would be, in those cases, no “specific intent” to modify germline cells.

The “Points to Consider” became Appendix M to the NIH “Guidelines for Research Involving Recombinant DNA Molecules.” As such, they created requirements for investigators funded by the NIH. However, the “Points to Consider” were never formally promulgated as federal regulations. The “Points to Consider” did not preempt federal, state, or local law. They also did not prohibit the conduct of germline gene transfer research that was privately funded, but merely offered the option of voluntary RAC review.

We should note that the oversight role of the RAC changed between 1984 and 2019. In 1996, the NIH and the U.S. Food and Drug Administration (FDA) agreed that the FDA would assume sole responsibility for the regulatory review of human gene transfer protocols. From 1996 forward, researchers could submit their protocols confidentially to the FDA while notifying NIH of their submissions. Between 1996 and 2019, the RAC continued to conduct public reviews of novel protocols. It also provided a public forum for the discussion of the serious adverse events that occurred in several gene transfer studies. In many ways, the RAC had evolved into a public advisory committee for the FDA.^25^

## The Legal Status of Germline Genome Editing in the United States

On April 28, 2015, NIH Director Francis S. Collins released a public statement on “NIH Funding of Research Using Gene-Editing Technologies in Human Embryos.”^26^ Dr. Collins based his opposition to germline genome editing on three arguments. First, the Dickey–Wicker Amendment “prohibits the use of appropriated funds for the creation of human embryos for research purposes or for research in which human embryos are destroyed” (H.R. 2880, Sec. 128). Second, “the NIH Guidelines state that the Recombinant DNA Advisory Committee ‘*will not at present entertain proposals for germ line alteration*’” (italics in original). Third, the FDA has the authority to regulate cell and gene therapy products, and the gene editing of human embryos cannot legally proceed in the United States without an Investigational New Drug (IND) application being in effect for the proposed research.

Dr. Collins's first argument lies beyond the scope of this article. However, we will briefly comment on the second and third arguments. The RAC and its working group did indeed make the statement that Dr. Collins quotes in 1985. However, in 2019, the RAC was disbanded, and there is currently no parallel public advisory body that fulfills the public protocol review role that the RAC played, at either the NIH or elsewhere in the U.S. federal government. The statement that the RAC will not review proposals therefore has no current practical application.

Regarding Dr. Collins's third argument, about the need for an IND review by the FDA, the legal situation in the United States is complicated. Since 2016, a rider to the federal appropriation bills that fund the FDA precludes the FDA from acknowledging receipt of an IND application that seeks to produce heritable genetic modifications in humans. The original target of the rider was the genetic modification of human embryos, but its effects spilled over to outlaw proposals for MR as well. In MR, nuclear DNA from the oocytes of a woman who is at high risk for transmitting mitochondrial disease to her offspring is combined with mitochondrial DNA from a donor woman whose mitochondrial DNA would not transmit the disease. MR can be carried out *in vitro* either before or after the fertilization of the recipient woman's oocytes.^27^ An alternative approach to achieving the same goal would be to perform *in vitro* genome editing on the mitochondrial DNA of the intending mother's oocytes.^28^ However, the use of either MR or mitochondrial genome editing for human reproduction would constitute a germline genome modification because it is heritable by matrilineal transmission from an affected woman. The net effect of the rider is thus to ban any attempt to perform genome editing in the reproductive context in the United States, including MR or the pre-fertilization editing of mitochondrial DNA.^29^

## Recent International and Intranational Discussions

The 2012 publication of a groundbreaking article by Emmanuelle Charpentier, Jennifer Doudna, and colleagues launched a new era in the history of human gene transfer.^30^ For the first time, genetic changes could be induced in cells in a more precise and targeted way using the CRISPR Cas-9 system. The National Library of Medicine recognized this advance when it added the term “gene editing” to its MeSH vocabulary in 2017. Professors Doudna and Charpentier received the 2020 Nobel Prize in Chemistry in recognition of their research.

The interest of scientists and the public in the promise of this new research tool has led to two international summits. The first meeting, the “International Summit on Human Gene Editing,” was held in Washington, DC, in December 2015.^31^ A subsequent meeting, the “Second International Summit on Genome Editing,” was held in Hong Kong in November 2018.^32^ A third summit is scheduled to occur in March 2022 and will be held in London.^33^

Beginning in 2015, public advisory groups have advanced the discussion of ethical, legal, and public policy issues in what is now customarily called “genome editing.”^34^ Of these reports and position statements, seven are particularly noteworthy and influential. In September 2016, the Nuffield Council on Bioethics published “Genome Editing: An Ethical Review.”^35^ This report was followed in February 2017 by a study from the National Academies of Sciences, Engineering, and Medicine (NASEM), “Human Genome Editing: Science, Ethics, and Governance.”^36^ In July 2018, the Nuffield Council on Bioethics published a second, more comprehensive, report entitled “Genome Editing and Human Reproduction: Social and Ethical Issues.”^37^ The German Ethics Council (Ethikrat) continued the public discussion in May 2019 with “Intervening in the Human Germline: Report” (“Eingriffe in die menschliche Keimbahn: Stellungnahme”).^38^ In 2020 and 2021, three major international reports on germline genome editing were published. In September 2020, the U.K. Royal Society and the U.S. Academies of Sciences and Medicine released “Heritable Human Genome Editing.”^5,39^ And in July 2021, the World Health Organization's Expert Advisory Committee on Developing Global Standards for Governance and Oversight of Human Genome Editing released two reports – regarding both the scientific aspects of this field.^40,41^

The Royal Society/NASEM report proposed a model for overseeing the clinical development of germline genome editing. This model is based on the oversight currently provided for MR by the U.K.'s Human Fertilisation and Embryology Authority.^5^

## A Plan for Public Oversight

**FIG. 1.**
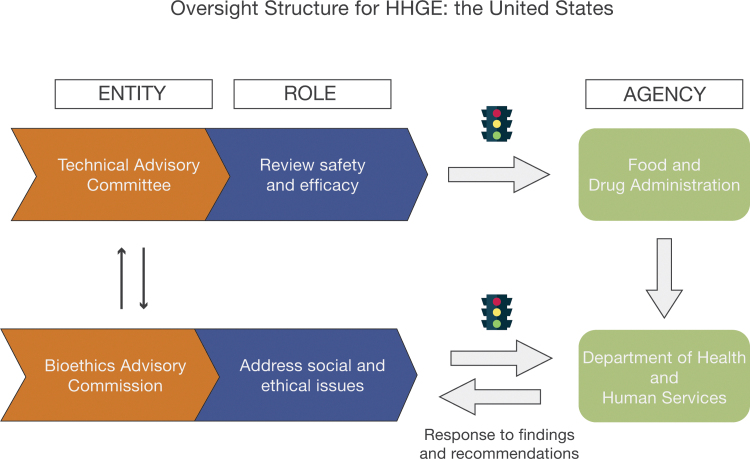
The complementary roles of the Technical Advisory Committee and the Bioethics Advisory Commission. This oversight structure comports with the recommendations of the WHO Expert Advisory Committee in its two recent reports. Graphic created by Adriane Inocencio at Arizona State University, edited by Michael Matason at Georgetown University. HHGE, heritable human genome editing.

Oversight of human gene transfer research by an advisory committee to the director of a funding agency (NIH) was in many ways a historical anomaly. A more reasonable approach to the regulation of germline genome editing in humans—an approach that builds on the RAC's experience—would include three components: (1) a technical review of the proposed research, (2) an evaluation of the social and ethical dimensions of that research, and (3) periodic international meetings that would summarize the global state of the art for genome editing. In the paragraphs that follow, we propose a three-part oversight structure for germline genome editing (see Fig. 1).

The first component of our proposed oversight structure would be regular public review of the state of the art in genome editing by an advisory committee to a regulatory agency. In the United States, that regulatory agency would be the FDA. We envision a Technical Advisory Committee (TAC) that would meet three times a year to review evidence about the safety and efficacy of somatic and germline genome editing. Meetings of the TAC would be held in public, and the FDA would have the authority to invite researchers to present both the challenges and successes of their recent studies. Specific topics to be addressed by the TAC closely parallel those identified by the Royal Society/National Academies report (see Supplementary Fig. S1):

- Evidence that genome editing is specific (hits the site intended and only that site);
- Evidence that genome editing works (percent success rate of alterations);
- Evidence of few or no off-target effects; and
- Evidence that the intended changes will produce the expected clinical outcomes.

The public meetings of the TAC would inform the FDA's confidential review of specific applications to perform research in humans that involves genome editing. As envisioned, this new advisory committee would perform a RAC-like role for genome editing.

A second critical component of effective oversight would be a national Bioethics Advisory Commission (BAC) for germline genome editing. Specific roles for the BAC would include:

- Mediating public engagement, with systematic “listening posts” attuned to the views of affected constituencies;
- Soliciting the perspectives of public interest advocates and religious and civic organizations;
- Reviewing and perhaps sponsoring, empirical social-scientific research to assess the degree of social consensus regarding germline genome editing in humans^42^; and
- Articulating criteria for determining when social consensus is sufficient to warrant the initiation of clinical germline genome editing protocols.

The BAC would report its findings to the Secretary of Health and Human Services (HHS), who would then transmit its advice to the FDA Commissioner. The findings of the commission would be published in the *Federal Register*. There would then be a 60-day period for public comments. Like the National Commission for the Protection of Human Subjects of the 1970s and the President's Commission on Bioethics of the early 1980s, the BAC would have response-forcing authority. That is, the Secretary of HHS would have a legal duty to respond to the commission's reports and recommendations and the public comments within 180 days of *Federal Register* publication. This commission should be authorized by the U.S. Congress and funded as a separate executive-branch agency to ensure its independence. Its members should be appointed by the President. The HHS Secretary should have final authority to approve or disapprove specific research protocols.

The work of the BAC would be informed by the reviews performed by the FDA's TAC and the confidential analyses and decisions of the regulatory agency regarding specific research applications. However, as noted above, the mandate of the BAC would be different. Its focus would be centered on the social and ethical implications of germline genome editing. Under this framework, a proposal to perform HHGE in a particular nation would require both the technical review of safety and efficacy for a particular protocol and the approval of that nation's bioethics advisory group.

A third and final component of our oversight proposal is a global forum that would meet every three to five years. At these public events, the scientific and public-policy developments of the intervening years could be systematically reviewed. The 2015 and 2018 international summits on human genome editing are examples of what can be achieved at such global meetings. In the proposed model, this global forum would collect and disseminate information, as well as inventory how different nations manage genome editing technologies. This international entity, logically sited at the World Health Organization or established as a collaboration of international organizations and academies, would facilitate information exchange. It would convene science experts, stakeholders, and civic action organizations, rather than having a regulatory role.

## Conclusion

In the late 1980s and the 1990s, the NIH RAC and its subcommittee created space for the innovative field of somatic cell human gene transfer. By today's standards, the methods employed for transferring genes in the 1980s and 1990s were relatively crude. The horizons opened by more precise techniques for genome editing have again raised a topic that the RAC decided to defer: HHGE. International meetings, committee reports, books, and articles from 2015 to the present have thoughtfully considered the technical, ethical, and public policy questions that methods for more precise germline genome editing raise. In this article, we propose a model for national public oversight of this important scientific arena.

## Supplementary Material

Supplemental data

Supplemental data
