# Supplementary material for: Governing Heritable Human Genome Editing: A Textual History and a Proposal for the Future
Source: CRISPR J. 2021 Aug 16;4(4):469–76. doi: 10.1089/crispr.2021.0043 (PMC8392078; doi:10.1089/crispr.2021.0043)

SUPPLEMENTARY INFORMATION

Attachment VII from the April 1, 1985, Meeting

of the RAC Working Group on Human Gene Therapy


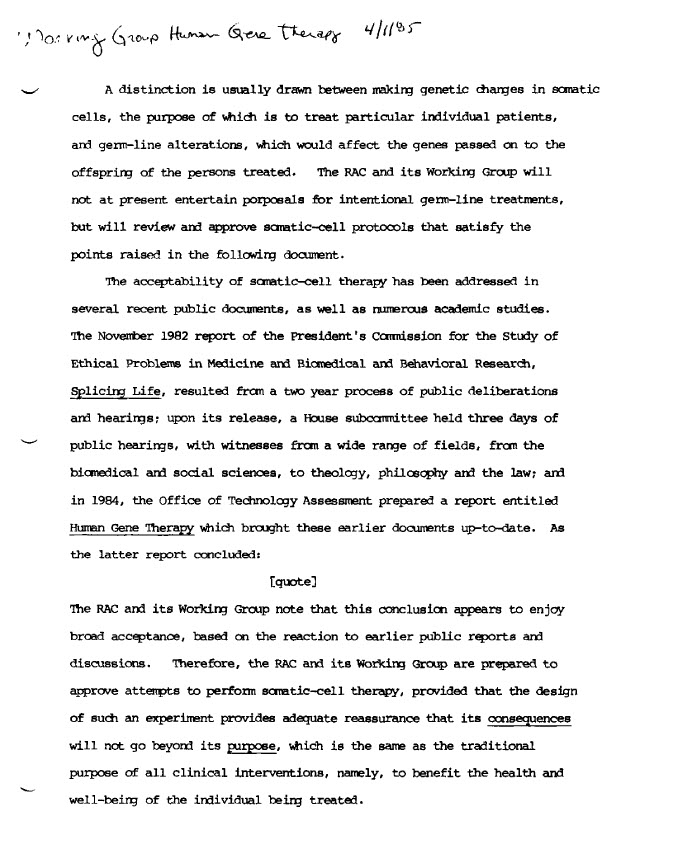

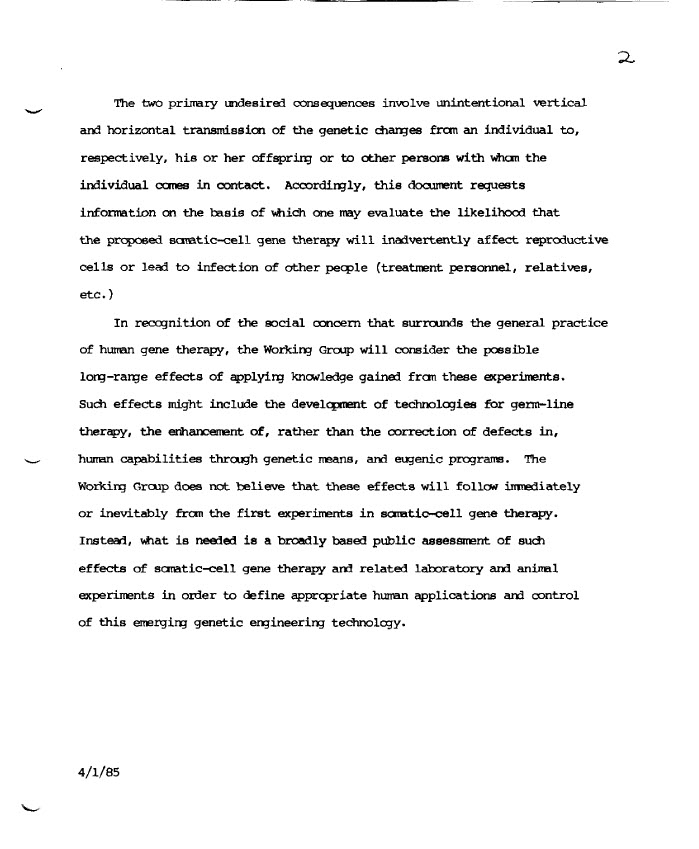


SUPPLEMENTARY INFORMATION

The April 15, 1985, Draft of the

“Points to Consider” Document


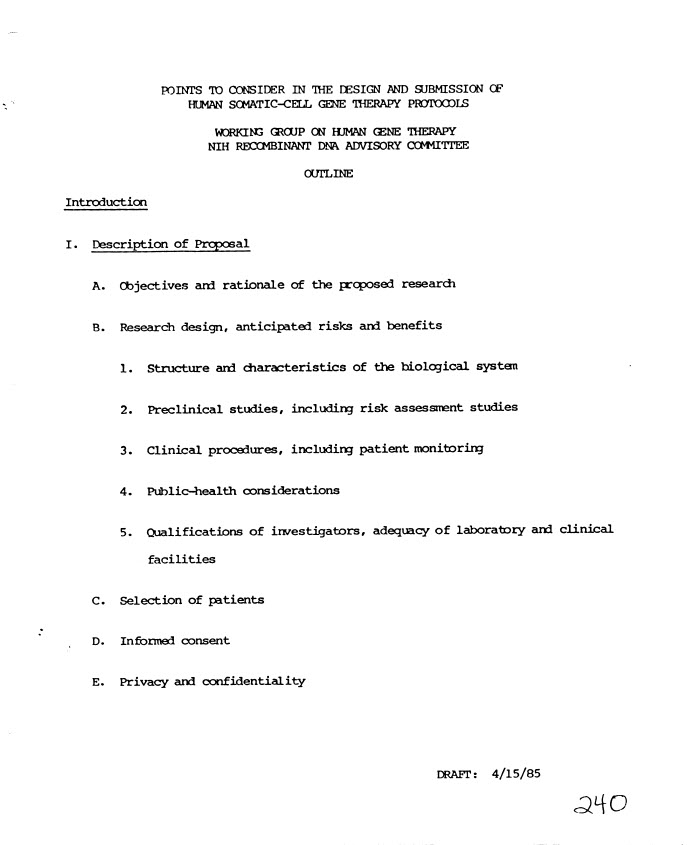


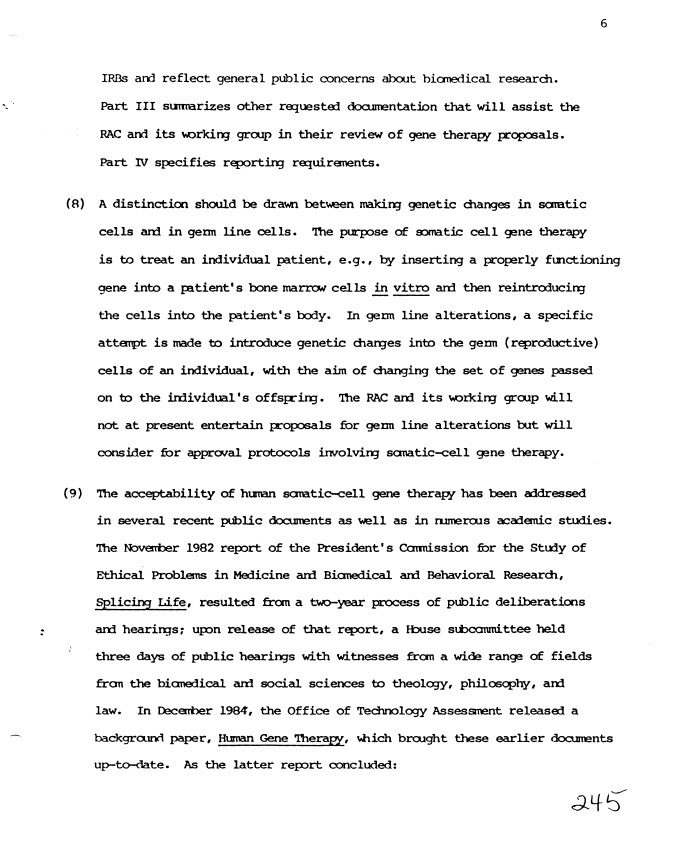


SUPPLEMENTARY INFORMATION

Minutes of the March 31, 1989, Meeting

of the RAC’s “Points to Consider” Subcommittee


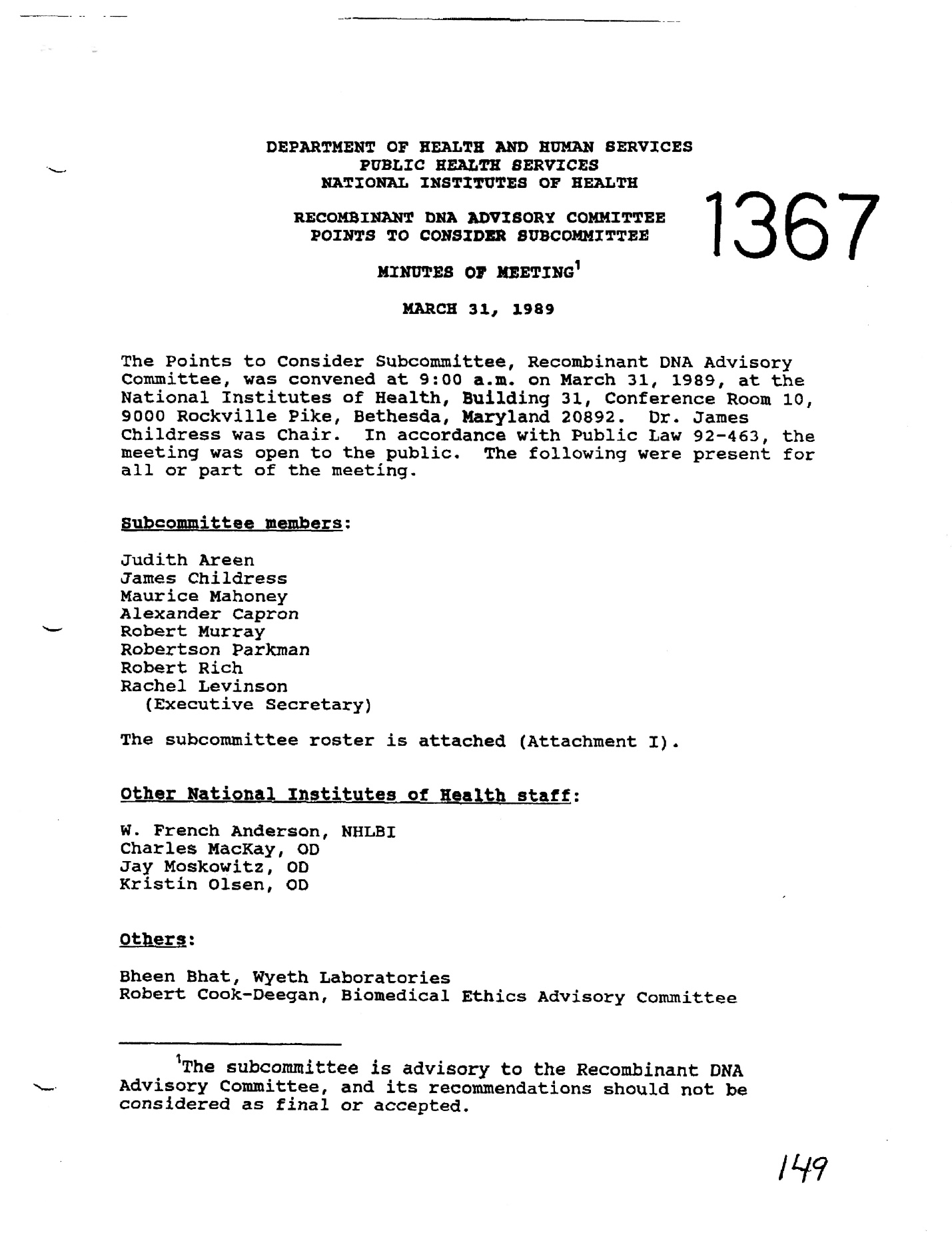


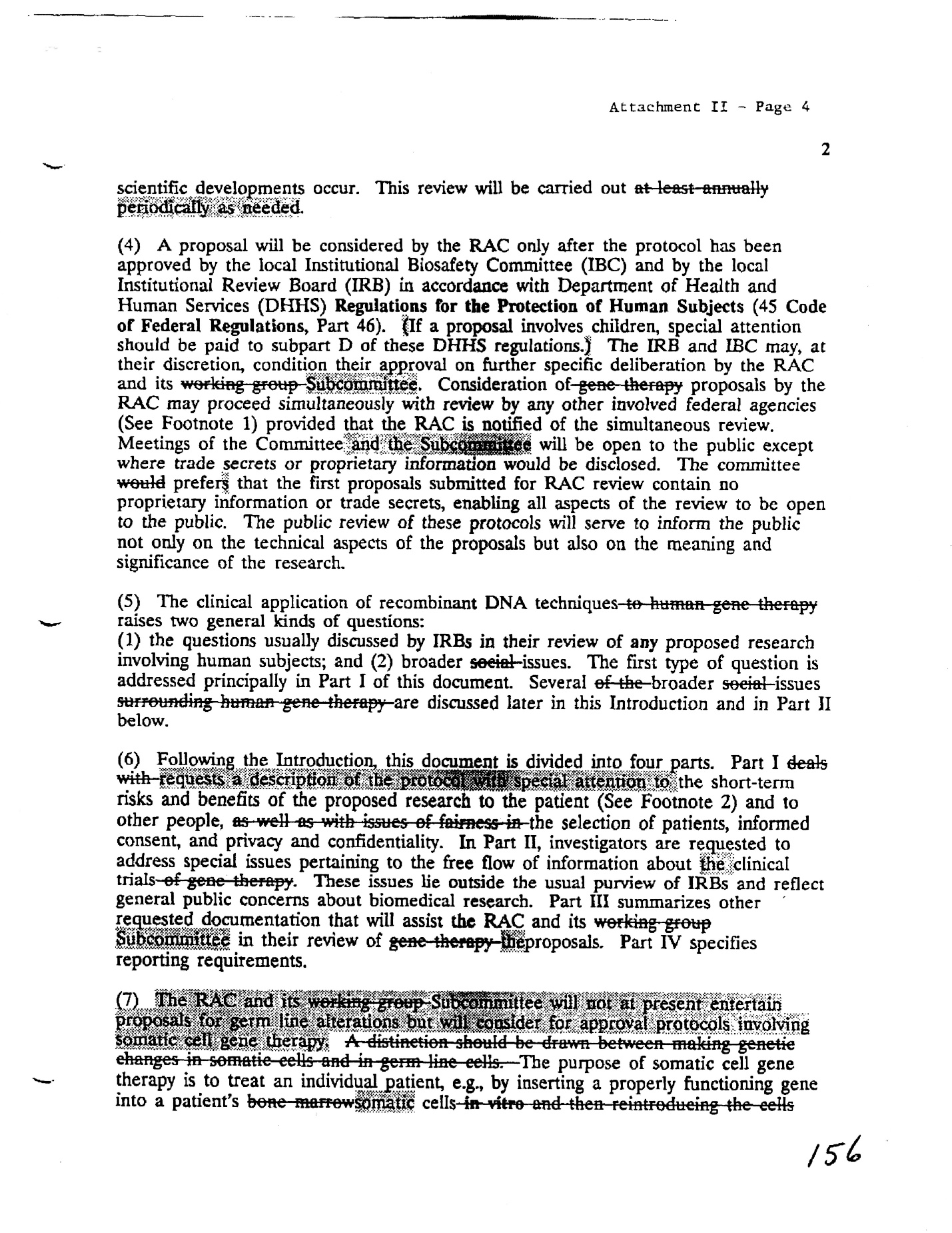


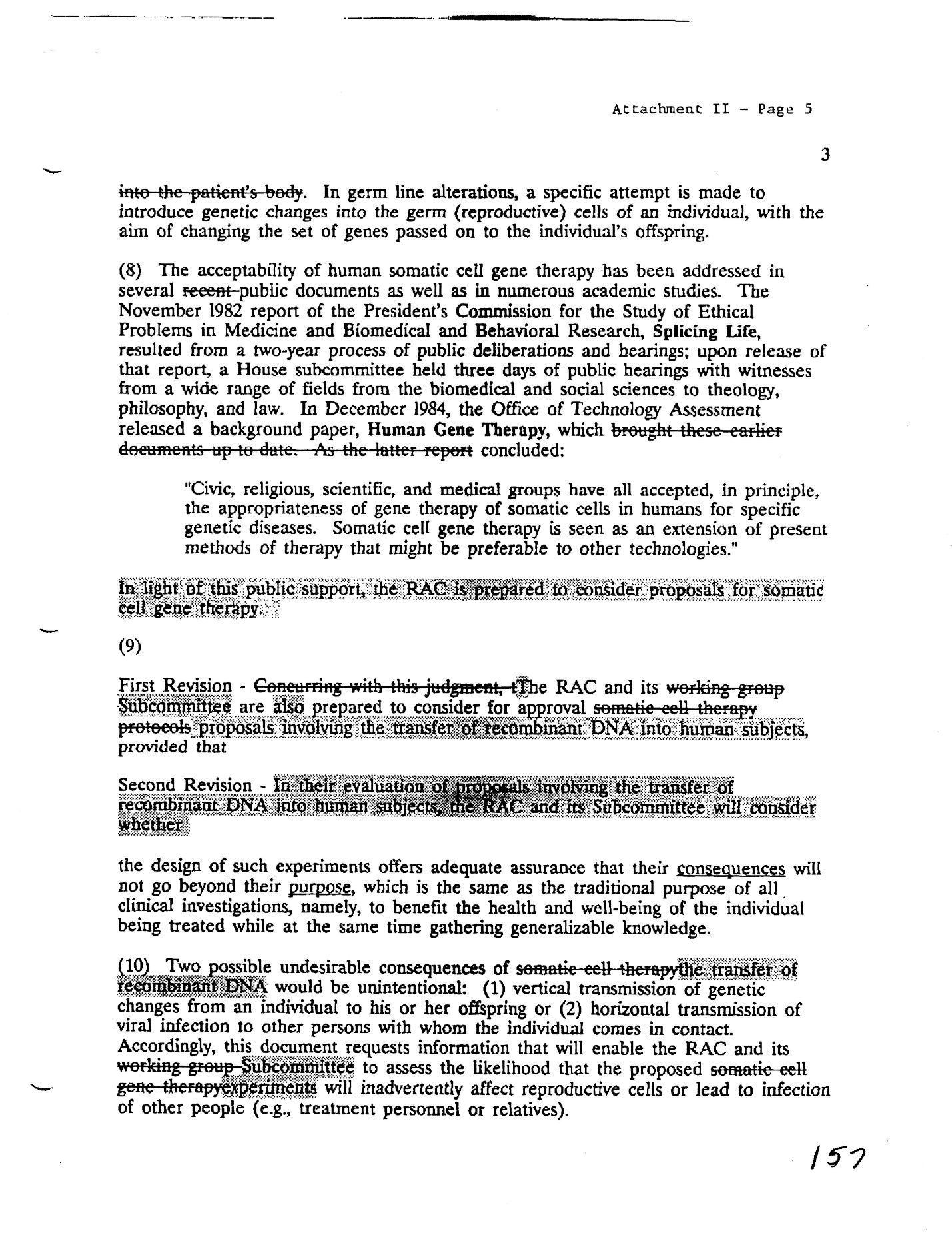

Supplement: Supplemental data [file Supp_Data.docx]
