# Supplementary material for: Governing Heritable Human Genome Editing: A Textual History and a Proposal for the Future
Source: CRISPR J. 2021 Aug 16;4(4):469–76. doi: 10.1089/crispr.2021.0043 (PMC8392078; doi:10.1089/crispr.2021.0043)
Supplement: Supplemental data [file Supp_FigS1.pdf]

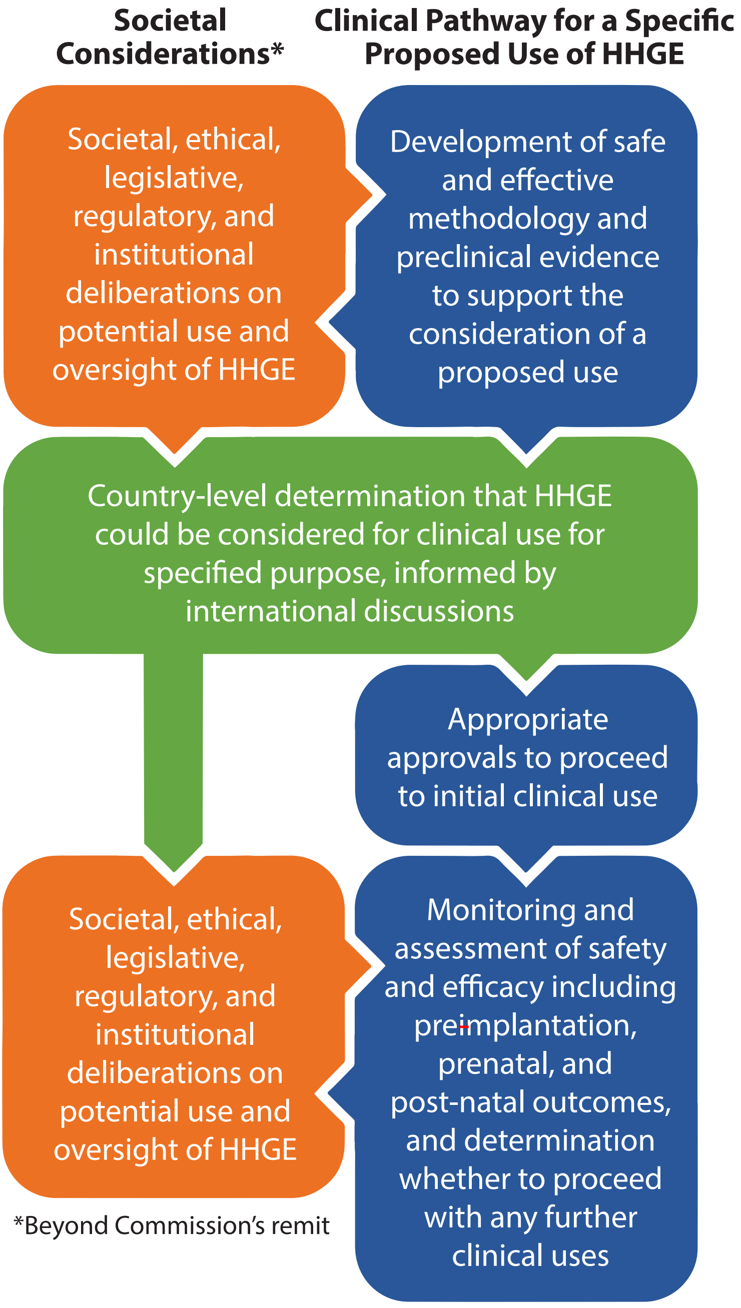
Societal Considerations*
Clinical Pathway for a Specific Proposed Use of HHGE
Societal, ethical, legislative, regulatory, and institutional deliberations on potential use and oversight of HHGE
Development of safe and effective methodology and preclinical evidence to support the consideration of a proposed use
Country-level determination that HHGE could be considered for clinical use for specified purpose, informed by international discussions
Appropriate approvals to proceed to initial clinical use
Societal, ethical, legislative, regulatory, and institutional deliberations on potential use and oversight of HHGE
Monitoring and assessment of safety and efficacy including preimplantation, prenatal, and post-natal outcomes, and determination whether to proceed with any further clinical uses
*Beyond Commission's remit
